# Enhanced rate capabilities in a glass-ceramic-derived sodium all-solid-state battery

**DOI:** 10.1038/s41598-020-66410-1

**Published:** 2020-06-11

**Authors:** Hideo Yamauchi, Junichi Ikejiri, Kei Tsunoda, Ayumu Tanaka, Fumio Sato, Tsuyoshi Honma, Takayuki Komatsu

**Affiliations:** 1 Nippon Electric Glass Co., Ltd., 7-1, Seiran 2-chome, Otsu, 520-8639 Shiga Japan; 2 Department of Materials Science and Technology, Nagaoka University of Technology, Kamitomioka-cho 1603-1, Nagaoka, Niigata 940-2188 Japan

**Keywords:** Inorganic chemistry, Batteries, Batteries, Phase transitions and critical phenomena, Structure of solids and liquids

## Abstract

An all-solid-state battery (ASSB) with a new structure based on glass-ceramic that forms Na_2_FeP_2_O_7_ (NFP) crystals, which functions as an active cathode material, is fabricated by integrating it with a β″-alumina solid electrolyte. Two important factors that influence the rate capability of this ASSB were optimised. First, the particle size of the precursor glass powder from which the NFP crystals are formed was decreased. Consequently, the onset temperature of crystallisation shifts to a lower temperature, which enables the softening of NFP crystals and their integration with β″-alumina at a low temperature, without the interdiffusion of different crystal phases or atoms. Second, the interface between the β″-alumina solid electrolyte and cathode active materials which consisted of the NFP-crystallised glass and acetylene black used as a conductive additive, is increased to increase the insertion/release of ions and electrons from the active material during charge/discharge processes. Thus, the internal resistance of the battery is reduced considerably to 120 Ω. Thus, an ASSB capable of rapid charge/discharge that can operate not only at room temperature (30 °C) but also at −20 °C is obtained. This technology is an innovative breakthrough in oxide-based ASSBs, considering that the internal resistance of liquid electrolyte-based Li-ion batteries and sulphide-based ASSBs is ~10 Ω.

## Introduction

In recent years, there has been significant development of lithium-ion batteries (LIBs) for use in small portable electronic devices but also in large electric vehicles. As a consequence of the increase in demand for energy-storage batteries with high capacities, new issues have arisen. First, there is concern regarding the supply of raw materials. In particular, the price fluctuations of lithium and cobalt, which are the raw materials of LIBs, have been considerable in recent years^1–5^. Second, heat management becomes difficult as the battery size increases, because heat dissipation from the surface becomes difficult. LIBs that use flammable organic electrolytes have issues with battery life and safety. Many next-generation batteries, that is, post-LIBs, have been studied with the aim of solving these problems. Sodium-ion batteries (SIBs) are considered to be potential post-LIB candidates.

As another alternative, all-solid-state batteries (ASSBs) with lithium^6–13^ or sodium^14–17^ as the guest ion have been proposed. ASSBs include an oxide solid electrolyte with a higher Young’s modulus than that of a sulphide solid electrolyte; as a result, it is difficult to reduce the interface resistance by pressure moulding alone^17^. However, because toxic hydrogen sulphide is not generated in oxide ASSBs, they can be considered as energy-storage devices with a low environmental impact; the environmental pollution is low not only during general use but also during their production. Although SIBs are attractive from the perspectives of abundance and cost, their energy density is lower than that of LIBs^9^. For this reason, it is important to develop novel materials that can be used to readily construct an ASSB with a bipolar structure^18^ by exploiting the high voltage resistance of an oxide-based solid electrolyte.

Our research group has previously reported the synthesis of different active materials for LIBs^19–26^ and SIBs^27–33^ via the crystallisation of phosphate glass using a melting method. In general, the crystallisation of glass proceeds via a continuous process of crystal nucleation and growth from a homogeneous inorganic matrix^34,35^. By optimising the composition of the matrix and the heat treatment process, the type of crystal that precipitates can be controlled in a single heat-treatment step, and the primary particle size can be nano- or microscale. Unlike conventional solid-state reactions, this method does not require pre-sintering and the crystallisation process is simple. In addition, when the precursor of glass is converted into a uniform melt at a high temperature, glass with uniformity at the atomic level is formed. Compared with the material obtained from a conventional solid-state reaction, this material can be regarded to have an extremely uniform matrix, which enables rapid and homogeneous formation of crystals.

Na_2_FeP_2_O_7_ (NFP) crystals, which function as the active material of the cathode in SIBs^36–40^, can be synthesised by the crystallisation of precursor glass using a melt quenching method^27–29^. We have previously utilised softening and flow in the glass transition region of 40Na_2_O‒20Fe_2_O_3_‒40P_2_O_5_ glass, which is the precursor glass of the NFP crystal and have succeeded in creating an ASSB through crystallisation with a reduction in the Fe ion content^41^. This oxide-based ASSB comprises NFP-crystallised glass as the active cathode material and a solid solution of β′′-alumina as the electrolyte. It was formed by coating a cathode paste on a solid electrolyte substrate, followed by heat treatment at 550 °C. An advantageous feature of this method is that the cathode‒solid electrolyte interface can be formed during this period without electrode pressurisation. The key phenomenon in the process of forming the interface is the softening and flow of glass that develops at the glass transition temperature and above. The reaction proceeds via nucleation and crystal growth from the glass matrix simultaneously with softening and flow. In a previous study^41^, the battery stably operated for 623 cycles at room temperature with a charge/discharge rate of 0.1 C. However, the evaluation was limited to 0.1 C because of the high internal resistance.

In this study, we used two approaches to control the formation of the active material–electrolyte interface in a Na-ASSB comprising NFP-crystallised glass to enable the operation of the ASSB at a higher current density. The first approach is to lower the crystallisation temperature to form a good conduction path that does not inhibit ionic conduction at the active material–electrolyte interface. The second approach is to increase the contact area of the interface between the active material, NFP, and the solid electrolyte, β″-alumina, or the conductive additive to enable instantaneous charge/discharge of a large current caused by the increase in the number of diffusion paths for Na ions and electrons in the NFP crystal. As ionic conductivity is proportional to the contact area, the resistance of the ASSB may be reduced by pulverising the glass powder or solid electrolyte powder used to form the cathode layer to increase the contact area. Alternatively, the contact area between the cathode layer and the solid electrolyte layer can be increased using a roughened β″-alumina substrate. In this study, we reduced the internal resistance by simply roughening the solid electrolyte‒electrode layer interface.

The ASSB developed in this study using the two approaches described above can be expected to show rapid charge/discharge at a large current because the input/output characteristics will be improved.

## Results and Discussion

### *In situ* impedance spectroscopy of the ASSB

**Figure 1:**
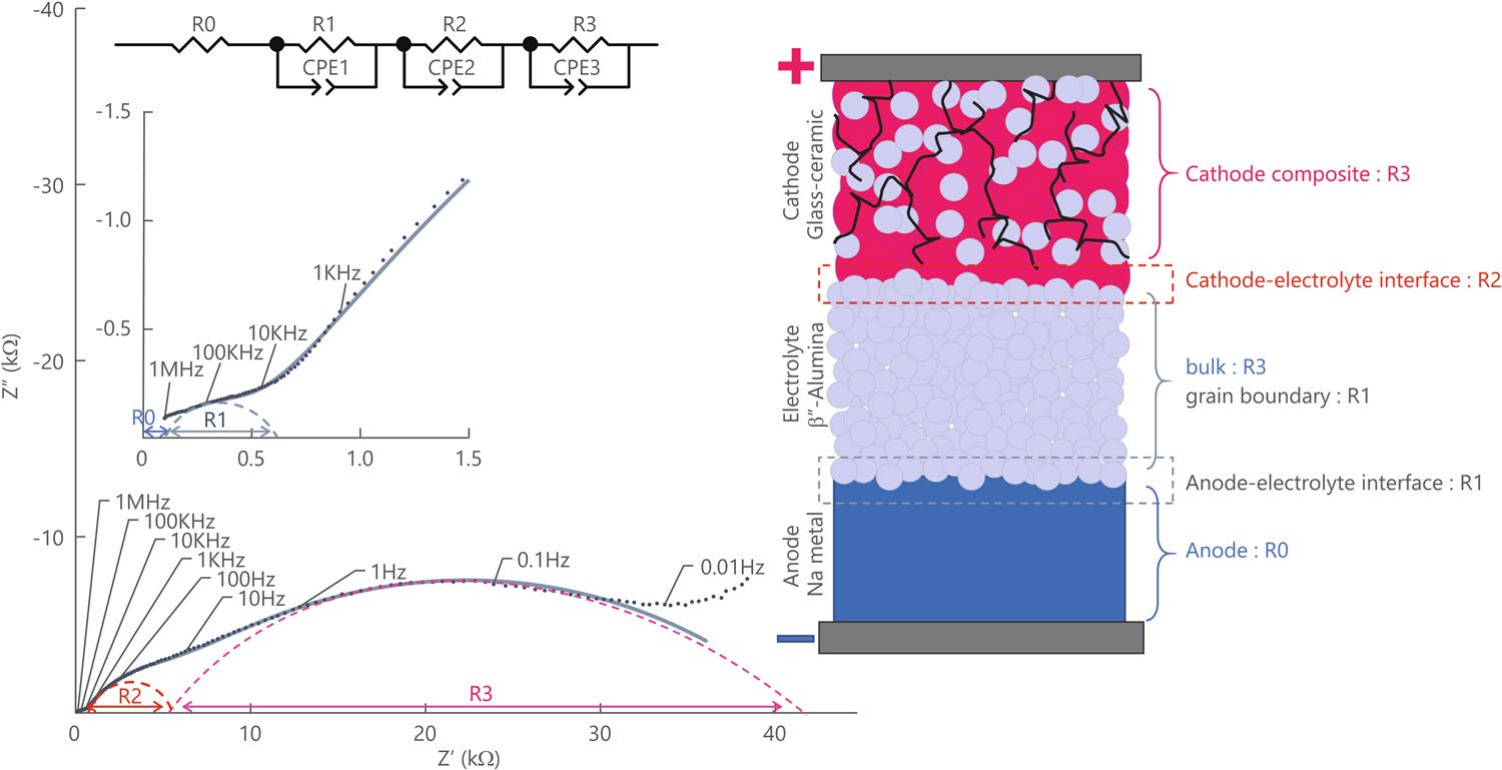
Room-temperature Nyquist plot and breakdown of each resistance component of an ASSB fabricated by conventional heat treatment.

To verify the effectiveness of the proposed approaches, we fabricated five ASSBs (Cells A–E). The heat-treatment conditions, cathode composition, and battery capacity of the fabricated cells are shown in Table S1 of the Supplementary Materials. Cell A was fabricated in the same manner as in a prior study^41^. To assess the effect of NFP glass particle size Cell B was fabricated using pulverised glass and β″-alumina and heat-treated for a shorter duration at a low temperature of 525 °C. Cell C was fabricated with an increased amount of the conductive additive acetylene black (AB). Cell D was calcined using a β″-alumina substrate with a roughened surface only the cathode side, and Cell E was calcined using a β”-alumina substrate with both surfaces roughened; details about this cell are discussed in the section “Porosity of the β″-alumina substrate and optimisation of the conductive additive.” Figure 1 shows a room-temperature Nyquist plot of Cell A, prepared under the same conditions as in a previous report^41^. Three kinds of semicircles, classified as large, medium, and small, were primary observed. They can be represented by equivalent circuits with charge transfer resistivities of R1, R2, and R3, respectively. The contribution of the resistivity of the β″-alumina substrate alone to the bulk resistance is as small as 2.7 Ω m (27 Ω with a 1 mm thick β″-alumina substrate), and this value is so small that it cannot be observed in the high-frequency region in Fig. 1. The major resistivity component R1 can be attributed to the charge-transfer resistivity of the anode–solid electrolyte interface. In the figure, the medium resistivity component R2 may be attributed to the interface between the cathode layer and solid electrolyte substrate (cathode–solid electrolyte interface), and the large resistivity component R3 originates from the resistivity of the interface of the NFP active material within the cathode mixture and solid electrolyte. The details of the attribution of resistivity components on a Nyquist plot of an all-solid-state battery are given in Table S2.

**Figure 2:**
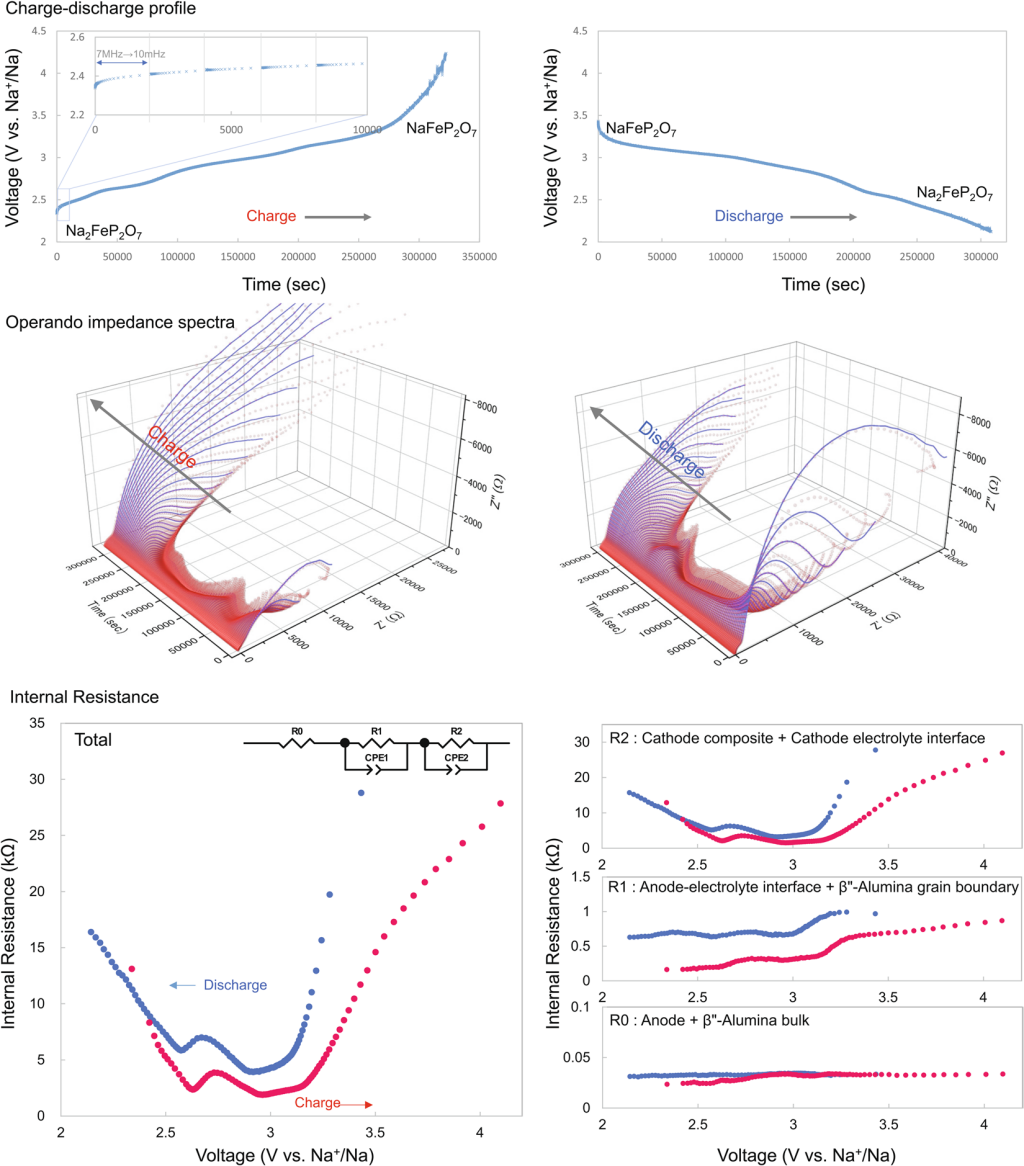
Charge/discharge profiles and Nyquist plots obtained by the *in situ* AC impedance method for an NFP-based ASSB, and change in the internal resistance for each component.

Impedance measurement, that is, “operando impedance spectroscopy,” was performed to examine these resistance components during the charge/discharge reaction^43,44^. The impedance data are continuously collected during the charge/discharge processes. Figure 2 shows the results of the operando impedance spectroscopy measurements of Cell A.

The results of impedance measurements of the newly prepared Cell A confirmed the existence of three semicircles. However, if the impedance is dynamically evaluated during charge/discharge, it would be difficult to distinguish it from the resistivity component R3, which is attributed to cathode–solid electrolyte interface, because the largest resistivity component R4, which is attributed to the cathode mixture, varied significantly. Therefore, the contributions from the cathode mixture and the cathode–solid electrolyte interface were treated as one combined resistivity component attributed to the “cathode,” and all the impedance curves of the cells were fitted with two semicircles. From the operando impedance measurements shown in Fig. 2, it can be seen that the internal resistance increases when the charge/discharge is completed. This large deviation that is dependent on the discharge depth arose from the charge-transfer resistivity, which is attributed to the “cathode.” This originated from the NFP crystal, which is the active cathode material during the charge/discharge process, due to the following reasons.

Na ions were released during the charging of the NFP crystal, which is the active cathode material. When it approached the end-of-charge voltage, the resistivity increased because of the release of Na ions from the interior of the active material particles, as well as due to the deficiency of Na ions that could be released. On the other hand, the resistivity increased during discharge because of the saturation of the Na ions inside the NFP crystals, since the ions occupied the space where Na ions could move.

Further, note that a two-step change at 2.6 and 3 V could be observed on the charge/discharge curve. The change at 3 V is considered to be due to a series of two-phase reactions and the change at 2.5 V can be attributed to a single-phase reaction of Na ions^40^. In the resistivity vs. voltage plot, a change in resistivity could be observed between 2.5 and 3 V where the reaction mechanism switched. Moreover, during the charge/discharge process, potential hysteresis was observed as is generally observed with cyclic voltammetry. These large values of resistivity arose from R2, which is attributed to the charge-transfer resistivity of the cathode mixture and cathode–solid electrolyte interface. Consequently, the reduction in the charge-transfer resistivity of the active cathode material is of great importance for improving the output characteristics of ASSBs.

Supplementary Fig. S1 shows the discharge-depth-dependence of the Nyquist plots of Cells A–E and a non-aqueous half-cell for reference. Supplementary Fig. S2 shows Nyquist plots at the discharge depth at which the impedance was lowest during the discharge process. The results for all the cells are consistent with the two semi-circular components shown in Fig. 1.

Thus, the internal resistance increased when the discharge was completed, and the resistivity of the active material depended on the depth of the discharge. When the guest ions saturated the crystal, the resistance increased in the active cathode material.

### Effect of the NFP glass particle size on the internal resistance

As for the crystallisation mechanism of NFP glass, we have shown that NFP crystallisation proceeds preferentially from the glass surface, that is, surface crystallisation occurs in the NFP glass and Na_2_MnP_2_O_7_ glass^30–33^. During the crystallisation, which proceeds from the glass surface, the glass experiences heterogeneous nucleation. The higher the number of nucleation sites formed the later the promotion of growth. That is, the crystallisation rate can probably be increased by decreasing the particle size and increasing the specific surface area of the glass. Increasing the crystallisation rate allows heat treatment to be applied at a lower temperature. The crystallinity of the glass showing this surface crystallisation is proportional to the specific surface area of the glass powder. Therefore, to complete the crystallisation at a lower temperature and over a shorter time, it is effective to use fine powder particles of an active cathode material and a solid electrolyte. Supplementary Fig. S3 shows the scanning electron microscopy (SEM) images of the NFP glass powder and β″-alumina powder. Fine powders of both NFP and β″-alumina solid solution were obtained by pulverisation. The specific surface area determined by the Brunauer–Emmett–Teller (BET) method is also shown in Fig. S3.

Supplementary Fig. S4(a) shows the differential thermal analysis (DTA) profiles of NFP glass powders of different particle sizes. It is clear that the exothermic peak accompanying crystallisation is shifted to a lower temperature as a result of pulverisation. It decreases by 54 °C from 491 °C to 437 °C. In addition, the crystallisation peak is sharpened. The sharp peak suggests that the surface crystallisation is completed within a narrow temperature range. This is because the crystallisation is facilitated by an increase in the specific area of glass powder due to the increase in the probability of crystallisation following the increase in temperature, as well as the number of nucleation sites on the surface. Through macro-type DTA measurements^45^, we were able to determine the temperature *T*_d_ (deformation point) at which the contraction of glass powder following sintering occurred, as well as the temperature *T*_s_ (softening point) at which softening and fluidisation of glass occurred. It can be seen in Fig. S4(a) that *T*_d_ and *T*_s_ were lowered by 39 °C as a result of pulverisation. From these results, it can be concluded that the increase in the surface area of the precursor particles due to the pulverisation of the powders favourably affected the softening and fluidisation of the powder, as well as the crystallisation of glass in terms of kinetics, thus making firing at a lower temperature possible.

The cathode layer comprises NFP-crystallised glass as the active cathode material, β″-alumina as the solid electrolyte, and AB as the conductive material. Supplementary Fig. S4(b) shows the DTA profile of this cathode layer. As expected, the crystallisation peak is shifted to a low temperature because of pulverisation.

Supplementary Fig. S5 shows the X-ray diffraction patterns of the cathode layer for each cathode composition. The crystalline phases of triclinic Na_3.12_Fe_2.44_(P_2_O_7_)_2_ and β″-alumina were confirmed and no other crystalline peaks were detected in the XRD patterns, suggesting an absence of impurities. These results contribute to a significant decrease in calcination temperature.

Supplementary Fig. S6 shows the SEM images and compositional profiles of the interface between the active material and the solid electrolyte in ASSBs calcinated at 550 and 525 °C. The presence or absence of bonding between the NFP and the β″-alumina is clear from the oxygen profile. Note that 550 °C for Cell A is the heat-treatment temperature applied in a prior study for Cell A^41^. It was confirmed that Na and P diffuse into β″-alumina at this temperature. Moreover, Cell B, which was heat-treated at 525 °C for a short duration of 30 min, was also well sintered and the diffusion of Na and P was suppressed. As a result, it was demonstrated that the interdiffusion of atoms at the solid–solid interface between the active material and the solid electrolyte can be suppressed by low-temperature/short-duration calcination.

**Figure 3:**
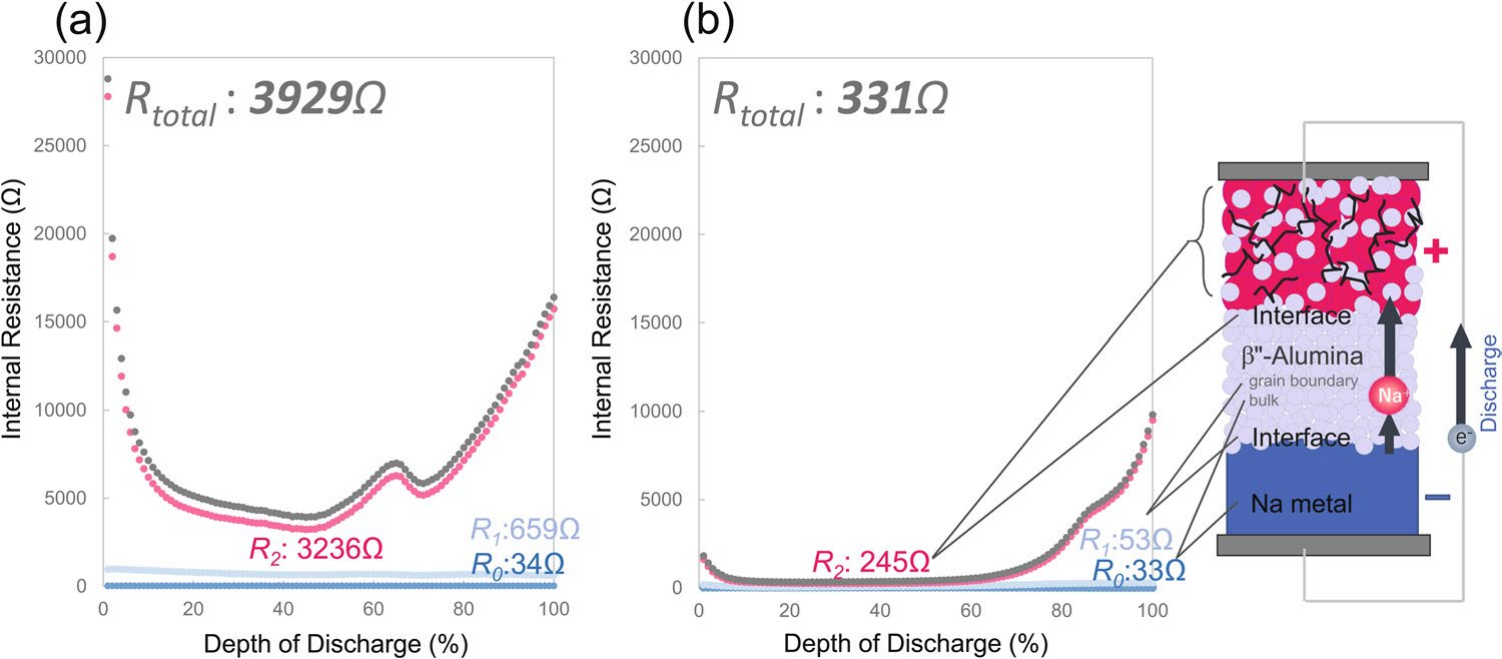
Charging state-dependence of each resistance component of Cells (**a**) A and (**b**) B produced using cathode active material powders of different particle sizes.

Figure 3 shows the discharge depth dependence of each resistance component. The peaks at 60% corresponds to the change in the charge/discharge mechanism discussed in Fig. 2. The resistance of the interface between the active material and the solid electrolyte is found to be significantly reduced by one  order of magnitude because of the pulverisation of the glass powder precursor and the heat treatment at a low temperature for a short duration. Therefore, the electrode layer can be formed at a low temperature over a short duration upon pulverising the NFP precursor glass powder used as the cathode material. This process successfully increased the number of conduction paths without inhibiting the ionic conduction between the active material and the solid electrolyte.

### Porosity of the β″-alumina substrate surface and optimization of the conductive additive

The resistance of the ASSB was decreased by decreasing the particle size of the NFP precursor glass and β″-alumina solid solution in the cathode layer. As shown in Fig. 3(b), there is room for improvement in suppressing the increase in resistance observed during the late stage of discharge. To this end, it is necessary to optimise the content of AB, which is a conductive additive. As the specific surface area increases as a result of the refinement of the NFP crystals and the β″-alumina, the proportion of AB, which becomes increasingly insufficient with increasing specific surface area, should be increased.

**Figure 4:**
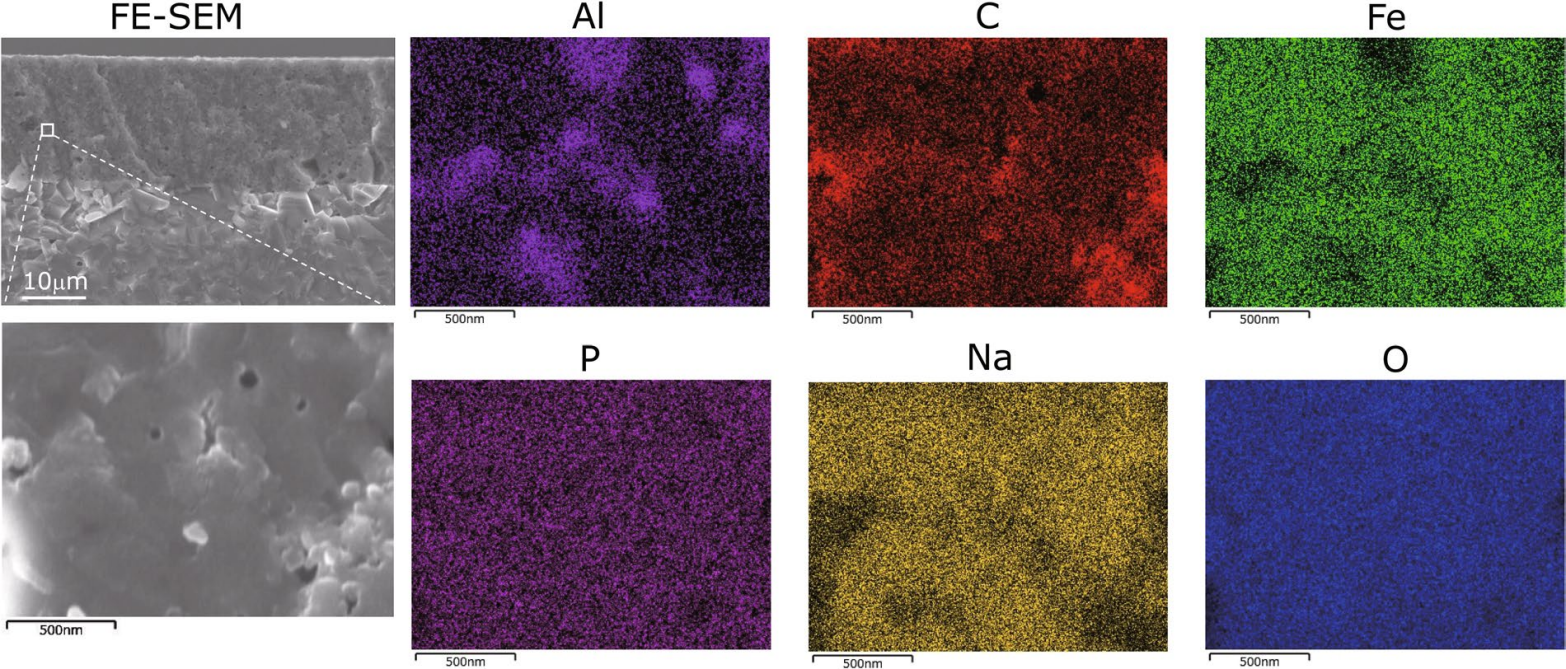
SEM images of the cathode layer and EDS maps of the constituent elements.

Figure 4 shows the SEM image and the energy-dispersive X-ray spectroscopy (EDS) maps of the constituent elements of the interface of the cathode layer‒β″-alumina substrate of Cell C. Cell C was prepared by increasing the content of AB from 3.5 to 4.2 wt% before calcination. The NFP, β″-alumina, and AB were found to be uniformly dispersed at the submicron scale in the cathode layer of Cell C. We further increased the AB content to 5 wt% and carried out calcination but not sintering. It is thought that the amount of NFP precursor glass have been insufficient. Therefore, the optimal ratio of the materials for the cathode layer in this study is possibly NFP:β″-alumina:AB = 83.4:12.4:4.2.

**Figure 5:**
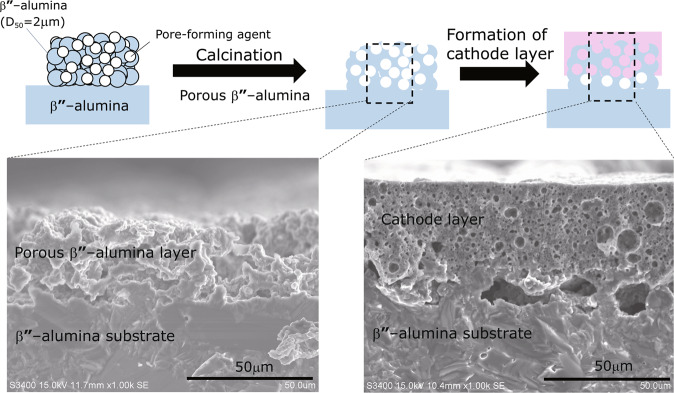
SEM images acquired after the formation of roughened β″-alumina substrate and cathode layer.

Furthermore, the use of a roughened β″-alumina substrate increased the contact area between the cathode layer and the alumina substrate, which is expected to decrease the internal resistance. Figure 5 shows cross-sectional SEM images of the roughened β″-alumina substrate and the bonding of the NFP layer. The surface was roughened using a pore-forming material. It is clear that the NFP has entered the pores. Although roughening the substrate effectively increases the contact area, it is also considered effective for the construction of a stronger physical bond at the interface via the anchor effect, which is observed in low-temperature cofired ceramics (LTCC). Peeling of the cathode material from the β″-alumina substrate because of its shrinkage during sintering can also be prevented by the anchor effect.

Kanamura *et al*.^8^ and Kotobuki *et al*.^42^ proposed a three-dimensional solid electrolyte ceramic using monodisperse polystyrene spheres as pore-forming agent to increase the contact area between the active material and the electrolyte. They applied this method to Li_4_Ti_5_O_12_ and reported a conductivity of 10^−5^ Scm^−1^ at room temperature. They also demonstrated the operation of an ASSB formed by a long-duration solid-state reaction with LiMn_2_O_4_ as the cathode layer at room temperature, although at a rate of only 1/100 C. Although a solid electrolyte with an arrangement of monodisperse pores contributes to an increase in the contact area, the structure and manufacturing process are likely to be complicated. However, this method is promising if a chemical reaction can suppress interactions between the active material and the solid electrolyte during heat treatment like this study.

**Figure 6:**
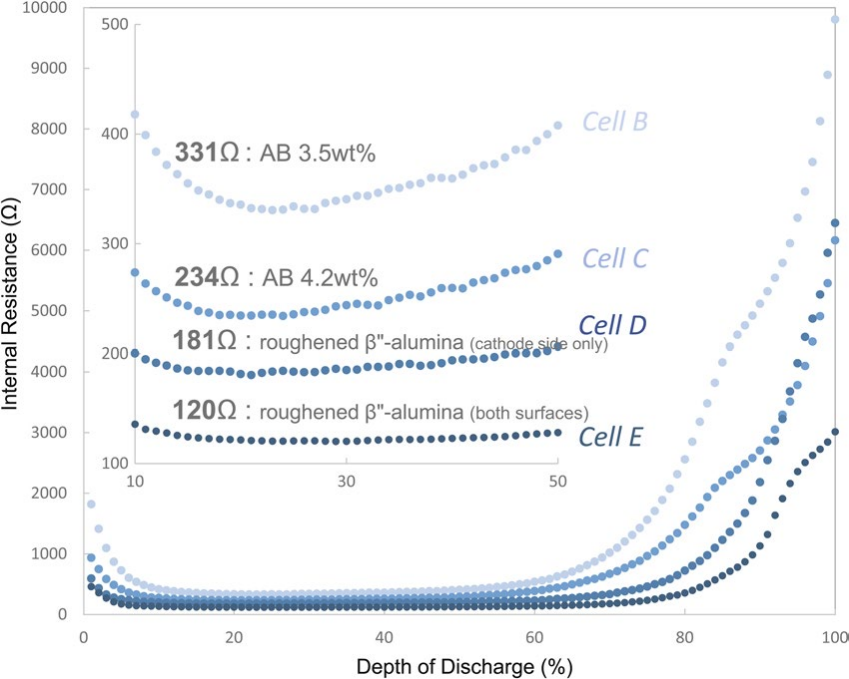
AB additive optimisation and discharge-depth-dependence of the internal resistance of batteries with roughened β″-alumina substrate.

As previously described, the internal resistance was further decreased by optimising the composition of the cathode layer and roughening the solid electrolyte substrate. Figure 6 shows the depth-dependence of the internal resistance during the discharge process for Cells B–E. The internal resistance of the cells was decreased from 331 Ω to 120 Ω with the optimisation of the conductive additive. In addition, by roughening the solid electrolyte used as a substrate, we reduced the internal resistance of the cell used in the previous study by ~30-fold from 3929 Ω to 120 Ω. This value rivals the internal resistance of sulphide-based ASSBs^12^. The electrical conductivity of β″-alumina is 10^−3^ S cm^−1^ at room temperature^46^ and the thickness of the β″-alumina substrate is 1 mm as in the present study. Therefore, there is room for further reduction in the internal resistance.

### Rate characteristics and low-temperature operation

**Figure 7:**
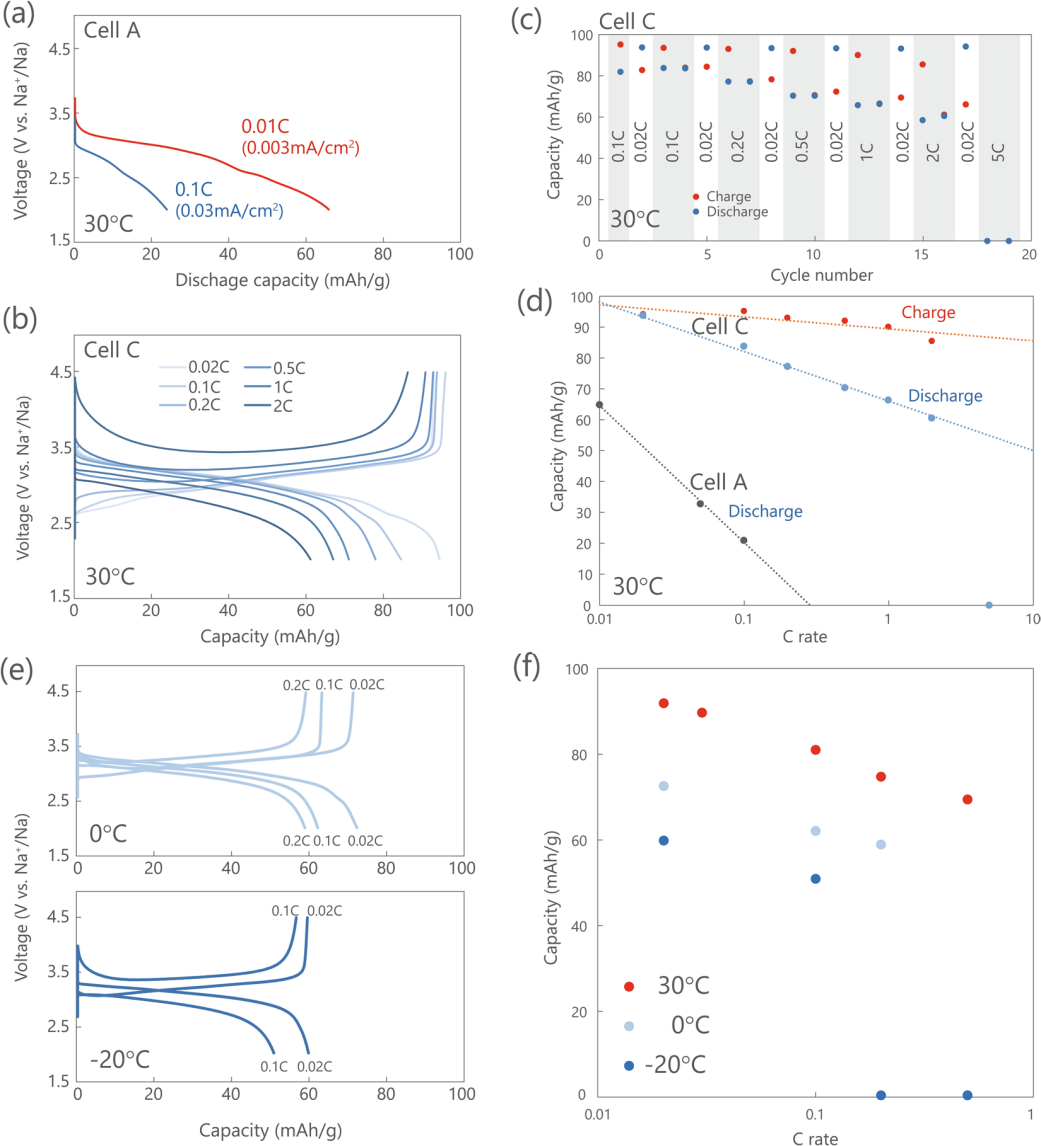
Rate characteristics of fabricated cells at each temperature: (**a**) Discharge profile of Cell A at 30 °C and 0.01 C and 0.1 C, (**b**) charge/discharge profile of Cell C at 30 °C, (**c**) cycle number of Cell C at 30 °C, (**d**) C rate-dependence of the charge/discharge capacity of Cells A and C at 30 °C, (**e**) charge/discharge profile of Cell C at lower temperatures, and **(f**) C rate-dependence of discharge capacity at each temperature.

The two different proposed approaches were effective for reducing the internal resistance of the oxide ASSB. As we have already demonstrated very good cyclic stability of the ASSB at room temperature in our prior studies, there is no concern regarding the battery life^41^. Accordingly, we focused on the rate characteristics. Figure 7(a–d) shows the charge/discharge profile and the rate-dependence of the charge/discharge capacity at room temperature at various charging rates (from 0.2 C to 2 C) of Cell C used as a representative cell, as its internal resistance was successfully reduced. For comparison, the results for Cell A are also shown. With the decrease in internal resistance, a discharge capacity of 61 mAh/g was obtained at 2 C, which corresponds to 63% of the theoretical capacity of 97 mAh/g. Previously a sulphide-based ASSB using LiNi_1/3_Co_1/3_Mn_1/3_O_2_ as the cathode was evaluated at room temperature, and its capacity has been reported to be approximately one-third of the discharge capacity at 0.15 C^12^. Although the battery configuration of the sulphide-based ASSB is completely different from that of our cells, if this value is considered to be the benchmark, the rate characteristics of Cell C are satisfactory and promising. Thus, by increasing the area of the interface through the pulverisation of the cathode material, we succeeded in increasing the number of diffusion paths of the ions and electrons, thereby realising a large current charge/discharge. Furthermore, the diffusion distance within the active material was also shortened by the pulverisation of the cathode material, which might be the reason for the improved input/output characteristics.

In addition, as shown in Fig. 7(d), the charging characteristics are very good and the charging capacity is 85.6 mAh/g at 2 C. This value is 88% of the theoretical capacity, indicating that rapid charging is realised. The reason for this result is that the internal resistance of the battery is 234 Ω during discharging, whereas it is 215 Ω during charging. Thus, it is considered that the NFP crystals show excellent performance in removing Na ions upon charging. In addition, the NFP cathode in ASSBs is stable enough to withstand overcharging by up to 9 V^41^, and safety can be guaranteed by increasing the cut-off voltage, even if the battery is charged more rapidly.

We further evaluated the charge/discharge characteristics at lower temperatures. Figure 7(e,f) shows the charge/discharge profiles and rate characteristics at 0 and −20 °C, respectively. A maximum discharge capacity of 58 mAh/g at 0.2 C was obtained at 0 °C, while a maximum discharge capacity of 50 mAh/g was obtained at 0.1 C at −20 °C. All cells were evaluated in a constant-current mode, and there is room to further increase this value by changing the charging process.

A pouch cell prototype with a cathode area of 25.2 cm^2^ based on Cell C (cathode area 1 cm^2^) was built to confirm the practicality of the cell. The area capacity of the cathode in the pouch cell was found to be 0.31 mAh/cm^2^, and the total pouch cell capacity was found to be 7.7 mAh. As shown in Supplementary Fig. S7, the minimum internal resistance for the discharge process of the pouch cell is 8.2 Ω and the sheet resistance is 206 Ω cm^2^, similar to that of Cell C (234 Ω).

**Figure 8:**
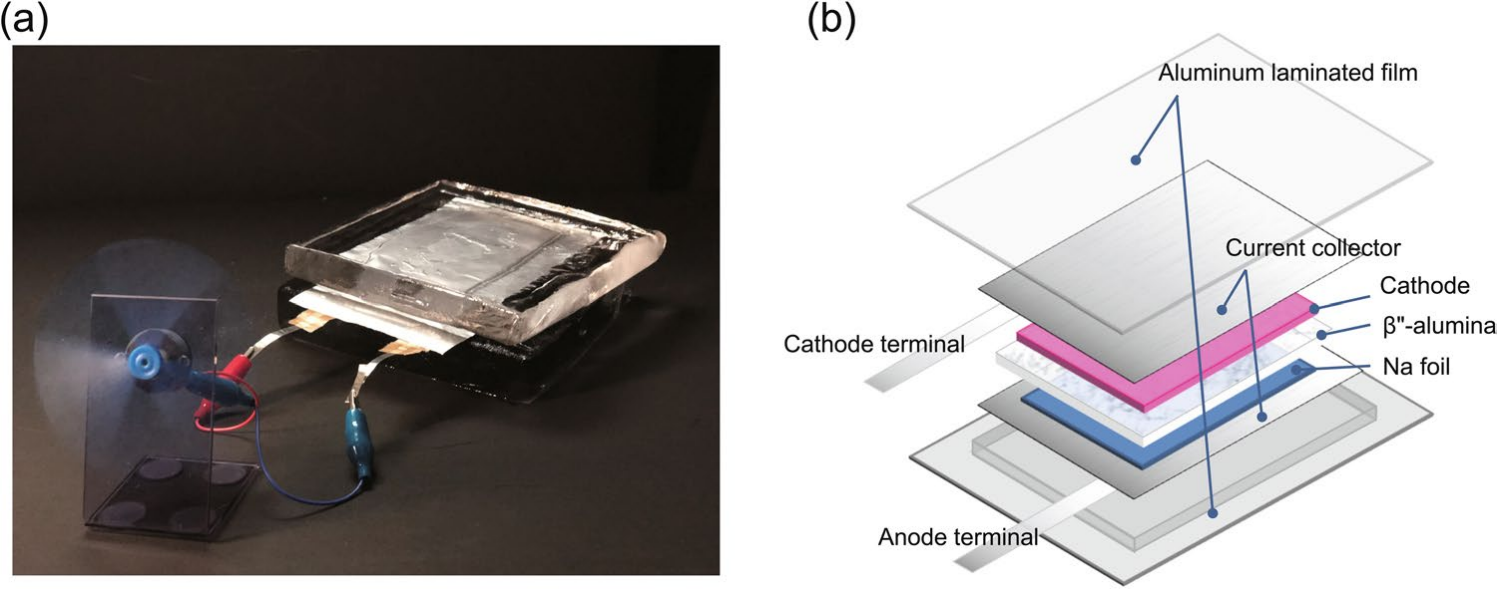
Motor operation test of the pouch cell based on Cell C at 0 °C: (**a**) optical image of an electric fan powered by the NFP glass-ceramic |β″-alumina|Na pouch cell (cell capacity 7.7 mAh and area capacity 0.31 mAh/cm^2^) sandwiched between two ice blocks, as shown in the Supplementary Video, (**b**) schematic illustration of the pouch cell.

Figure 8 and Supplementary Video 1 demonstrate the operation of the pouch cell at 0 °C. It can be seen that the motor operates even below the freezing point of water. Thus, our Na-ASSB can be expected to operate in cold regions owing to its low resistance. This is a highly significant result because the oxide solid electrolyte has low ionic conductivity^47^ and it is difficult to operate an oxide ASSB at room temperature^48^ owing to the formation of a heterogeneous layer at the interface between the solid electrolyte and the active electrode material during the calcination process^49^.

Kato *et al*. have reported that LIBs with a sulphide electrolyte or a liquid electrolyte have excellent output characteristics because their internal resistances are about 10 Ω^9^. We have succeeded in reducing the internal resistance of an oxide-based Na-ASSB and clarified that its characteristics are comparable to those of a sulphide-based Li-ASSB. The relationship between the rate characteristics and internal resistance is summarised in Table 1, from which it is apparent that the discharge capacity at a high rate improves as the internal resistance decreases. This is the first report of an oxide-based Na-ASSB with such good characteristics. Therefore, the rate characteristics shown in Fig. 7 are likely to be used as future benchmarks.

**Table 1:** Rate capability and internal resistance of ASSBs.

Cell	Discharge capacity (mAh/g)										Internal resistance(Ω)
	0.01 C	0.02 C	0.05 C	0.1 C	0.2 C	0.5 C	1 C	2 C	5 C	10 C	
A	66	—	33	21	—	—	—	—	—	—	3929
B	—	90.9	—	83.5	—	64.9	0	—	—	—	331
C	—	93.9	—	83.9	—	70.5	66.4	60.6	0	—	234
D	—	88.4	—	81.4	72.9	65	58.9	50.2	27.1	0	181
E	—	94.9	—	80.0	70.4	61.1	56.1	46.9	30.8	15.3	120
1M-NaPF_6_ in EC:DEC	—	—	—	89.0	83.1	76.9	71.5	62.4	35.6	5.8	138

## Conclusion

We proposed a new approach to enhance the rate characteristics of an oxide ASSB comprising a β″-alumina solid electrolyte and NFP-crystallised glass as the active cathode material. In this approach, we decreased the particle size of the precursor glass powder to promote the crystallisation of NFP crystals from the glass powder surface. Consequently, the onset temperature of crystallisation shifted to a lower temperature, enabling the softening of NFP crystals and their integration with β″ alumina at a low temperature. Consequently, a good ion conduction path was formed in the electrode. Further, we increased the interface between the cathode, solid electrolyte, and the conductive additive to increase the insertion/release of ions and electrons from the active material. An internal resistance rivalling that of a sulphide-based ASSB was achieved by optimising the blending ratio of the constituents of the cathode layer and increasing the area of the interface between the active cathode material and the β″-alumina solid electrolyte or conductive additive. Thus, we demonstrate that it is no longer difficult to decrease the internal resistance of oxide ASSBs, which would enable further research leading to practical application. The approach used here is only one example of various means of enhancing the rate characteristics of an ASSB, and we are investigating other ways to improve the characteristics.

## Methods

### Glass production

The glass was produced by conventional melt-quenching process according to a previously published method^27,28^. Sodium metaphosphate (NaPO_3_), ferric oxide (Fe_2_O_3_), and orthophosphoric acid (H_3_PO_4_) were used as raw materials. A powder precursor was prepared with the composition of 40% Na_2_O, 20% Fe_2_O_3_, and 40% P_2_O_5_. This powder was melted in air at 1250 °C for 45 min in a Pt crucible. Thereafter, the melt was poured between twin rollers and glass was formed by rapidly cooling the melt to obtain glass flakes with thicknesses ranging from 0.1 to 1 mm. The so-obtained film-like glass was dry-milled using a ball mill to obtain a coarse glass powder, which was further pulverised using a planetary ball mill (Pulverisette 6 manufactured by Fritsch) to obtain the glass powder (precursor glass powder of the active cathode material) described in Table S1.

### Preparation of β″-alumina solid solution

Solid electrolyte, β″-alumina, substrate was prepared by conventional solid-state-reaction process. The powder was obtained by roughly pulverising commercial β″-alumina (manufactured by Ionotec) first with an agate mortar and pestle, and then with a ball mill and a planetary ball mill. The characteristics of the solid electrolyte powder are presented in Table S1. The green-body was then calcined at 1600 °C for 1 h to obtaine sintered body with a size of 12 mm × 12 mm × 1 mm.

For the preparation of roughened β″-alumina substrte, the solid electrolyte powder with a specific surface area of 5 m^2^ g^−1^ (average particle size D_50_ of 2 μm) was used. Acrylic polymer particles (ADVANCELL HB2051 manufactured by Sekisui Chemical; average particle size of 20 μm) were mixed with the solid electrolyte at a solid electrolyte:polymer particle volume ratio of 25:75, and 20 wt% polypropylene carbonate (Empower Materials) was added as a binder to 100 parts by mass of the mixture. After that, the mixture was dispersed in N-methylpyrrolidone and stirred well using a rotation/revolution mixer to form a slurry. The obtained slurry was applied to the β”-alumina substrate to a thickness of 100 μm using a doctor blade and then dried at 70 °C. This was then reheated at 1600 °C for 1 h to produce a porous solid electrolyte.

### Production of the electrode mixture

The cathode active cathode material precursor powder (NFP-crystallised glass) and solid electrolyte powder (β″-alumina), obtained as previously described, and AB (SUPER C65 manufactured by TIMCAL) as a source of conductive carbon, were weighed in the proportions described in Table S1, and 10 wt% propylene carbonate (Empower Materials) was added to the 100 wt% powder. An additional 30 wt% N-methylpyrrolidone (Kishida Chemical) was added and the mixture was stirred well using a rotating/revolving mixer (THINKY ARE-310) to form a slurry. The obtained slurry was applied to one surface of the solid electrolyte layer to with an area of 1 cm^2^ (for Cells A–D) or 25.2 cm^2^ (for the pouch cell) to a thickness of 100 μm, and then dried at 70 °C for 3 h.

### Formation of the cathode layer (calcination)

A cathode layer was formed on one surface of the solid electrolyte layer by placing the aforementioned layer in a carbon container and calcinating it in H_2_/N_2_ (H_2_/N_2_ = 4/96 v/v%) atmosphere under the conditions described in Table S1. All the aforementioned operations were performed in an environment with a dew point of −40 °C or lower.

### Production of ASSBs

The ASSBs were produced according to a previously published method^41^. A current collector composed of an Al electrode with a thickness of 300 nm was formed on the surface of the cathode layer using a DC magnetron sputtering apparatus. Thereafter, CR2032-type test batteries (Cells A–E) were produced by pressure-bonding metal sodium as the counter electrode to the other surface of the solid electrolyte layer in argon atmosphere at a dew point of −60 °C or lower. Then, the battery was placed on the lower cap of a coin cell, and then covered with an upper lid. A pouch cell based on Cell C was prepared by pressure-bonding metal sodium on the counter electrode in the same manner. An aluminium foil was used as a current collector and the aluminium laminate films were heat-sealed to form a package.

### Fabrication of a battery with a non-aqueous electrolyte

Crystalline glass powder with an average particle size of 2 μm was obtained by pulverising the crystallised glass, the aforementioned film-like glass crystallised at 650 °C, with a ball mill and then air-classifying. To 80 wt% of the crystallised glass powder, 20 wt% of polyethylene oxide nonyl phenyl ether (mass-average molecular weight: 660), which is a non-ionic surfactant, was added as a source of carbon, and the mixture was mixed well. Subsequently, the non-ionic surfactant was carbonised by calcining the mixture at 600 °C for 1 h under a nitrogen atmosphere to obtain a pulverised active cathode material covered with carbon. The powder X-ray diffraction pattern of the cathode material showed diffraction lines originating from Na_3.12_Fe_2.44_(P_2_O_7_)_2_ crystals.

For the preparation of the cathode, AB and polyvinylidene difluoride (PVDF) as the conductive additive and binding agent, respectively, were weighed according to Table S1. Subsequently, after dispersing them in N-methylpyrrolidone, a slurry was formed by blending the mixture with a planetary centrifugal mixer. After that, the obtained slurry was applied onto 20 μm thick Al foil that serves as a cathode current collector, using an applicator with a gap width of 150 μm. After drying at 70 °C with a dryer, it was passed through a pair of rollers, and then pressed at 1 t/cm^2^ to form an electrode sheet. A circular working electrode was obtained by punching the electrode sheet into a circle of 11 mm diameter with an electrode punching machine, and then drying it at 140 °C for 6 h. The obtained working electrode was then placed on the lower lid of a coin cell, with the Al foil side facing downwards. A glass filter dried at 200 °C for 8 h and a separator consisting of a polypropylene porous membrane (Celgard #2400, Celgard) with a diameter of 16 mm, which was dried under reduced pressure at 60 °C for 8 h, as well as metallic sodium as a counter electrode, were laminated on top of the working electrode. Thus, the CR2032 type test battery was fabricated. A 1 M NaPF_6_ solution in EC: DEC = 1: 1 (EC = ethylene carbonate, DEC = diethyl carbonate) was used as the electrolyte solution. The assembly of the test battery was performed under an argon atmosphere below the dew-point temperature of 70 °C.

### Thermophysical analysis

Thermophysical properties were evaluated by DTA (DTA8410, manufactured by Rigaku) using an Al cell with a sample weight of 300 mg at a heating rate of 3 °C/min under the flow of a H_2_/N_2_ gas mixture (H_2_/N_2_ = 4/96 v/v%) at a rate of 0.1 L/min.

### SEM-EDX

A Hitachi High-Technologies SU8220 scanning electron microscope was used for the FE-SEM observations. A HORIBA EMAX Evolution EX-370/X-Max150 system was used for EDS. The calcination of the cathode mixture, NFP glass (27 m^2^g^−1^, 0.2 μm):β″-alumina (45 m^2^g^−1^, 0.1 μm):AB = 83.4:12.4:4.2 wt% was performed at 525 °C for 30 min in H_2_/N_2_ mixed gas (H_2_/N_2_ = 4/96 v/v%) atmosphere on the solid electrolyte substrate. The joined cathode‒solid electrolyte was cut in a dry room and then observed.

### Assignment of each component in Nyquist plot

AC impedance measurement was conducted on Cell A with the applied voltage of 5 mV in the frequency region of 1 MHz to 25 mHz at 30 °C at the open circuit voltage using VersaSTAT4 (Princeton Applied Research). The Nyquist plot was analysed as described below, and the results are summarised in Table S2.

First, for each material and composite that constitutes an ASSB, the components of resistivity were analysed through AC impedance measurements at 30 °C with the applied voltage of 5 mV in the frequency range of 10 MHz to 10 mHz, using Solatron 1260 A (Solatron Analytical).

Another AC impedance measurement was then performed to investigate the resistivities of the cathode–solid electrolyte interface and anode–solid electrolyte interface, with the fabricated cathode composite/β″-alumina/cathode composite as the cathode symmetric cell and Na/β″-alumina/Na as the anode symmetric cell. An equivalent circuit was created using Z-ASSIST (software fabricated by TOYO Corporation) based on the obtained Nyquist plot, and the value of each resistivity component of the equivalent circuit was obtained by fitting the Nyquist plot using Zview (Scribner Associates Inc).

### Operand AC impedance

Galvanostatic electrochemical impedance spectroscopy (GEIS) measurements were repeated for each prototype battery with constant-current charging at 30 °C using a VMP-300 system (Bio-Logic Science Instruments). The impedance spectrum was obtained up to 4.5 V. Similarly, GEIS measurements were repeated up to 2.0 V while discharging the battery at a constant current and an impedance spectrum was obtained (red dots in Fig. S1). Using Z-3D software (produced by Toyo Corporation), we spline-interpolated the obtained result to divide either the charging or discharging measurement interval into 100 such that a Nyquist plot at a specific time could be extracted (blue lines in Fig. S1). An equivalent circuit for the Nyquist plot calculated with Z-3D was created using Z-ASSIST software (produced by Toyo Corporation). Using this equivalent circuit, we fitted the Nyquist plot by Zview (Scribner Associates) to determine the value of each resistance component of the equivalent circuit. Using Z-FIT-Analysis software (produced by Toyo Corporation), we applied the equivalent circuit to each spectrum in the Nyquist plot calculated using Z-3D, and the change in internal resistance was obtained by collectively performing fitting analysis. The plots in Figs. 3 and 6 that show the change in internal resistance with respect to the depth of discharge are the results of plotting this resistance at intervals of 1%.

Supplementary Fig. S2 shows Nyquist plots indicating the lowest total internal resistance during the discharge process. The strongest correlation with the rate characteristic can be found for this internal resistance. The resistance of the resistivity component R2, which corresponds to a capacitive semicircle, varied significantly depending on the change in the discharge depth, and it could be attributed to components including the charge-transfer resistivity of the composite cathode material and the charge-transfer resistivity of the cathode–solid electrolyte interface. R1 could be attributed to the resistivities of the anode–solid electrolyte interface and the grain boundary of the solid electrolyte. R0 could be attributed to the resistivity of the anode and the bulk (inside a crystal grain) resistivity of the solid electrolyte.

Since the charge transfer resistivity R2 of the cathode is considerably large for Cell A, we speculated that the charge transfer resistivity R1 of the interface of the counter electrode (anode) also became large. For Cell B, since the charge transfer resistivity R2, which originates from the cathode was reduced by one  order of magnitude, R1 was also reduced by one  order of magnitude. Due to the increase in the area of the cathode–solid electrolyte interface due to the roughening of the substrate surface on the cathode side in Cell D, it can be understood that the charge transfer resistivity of R2 was reduced since the charge transfer resistivity of the cathode–solid electrolyte interface had decreased. Furthermore, for Cell E, the resistance of R1 was reduced owing to the roughening of the anode–solid electrolyte surface in addition to the roughening of the cathode. To summarise, it can be concluded that each charge transfer resistivity R1 and R2 of the cathode and anode, respectively, were complementarily correlated.

### X-ray diffraction measurement

To determine the crystalline phase of the cathode layer, X-ray diffraction patterns were recorded with a RINT-2100 diffractometer (Rigaku) using Cu-Kα radiation. Supplementary Fig. S5 shows the X-ray diffraction patterns of the cathode composite materials calcined at 550 °C for 60 min (Cell A) and 525 °C for 30 min (Cells B and C) on a β″-alumina solid electrolyte substrate in H_2_/N_2_ (H_2_/N_2_ = 4/96 v/v%). Excluding the diffraction signals from β″-alumina, only the diffraction signals from triclinic Na_3.12_Fe_2.44_(P_2_O_7_)_2_ were observed for the cathode layers of Cells A–C.

## Supplementary information

Supplementary information.

Supplementary information2.
