# Supplementary material for: Enhanced rate capabilities in a glass-ceramic-derived sodium all-solid-state battery
Source: Sci Rep. 2020 Jun 11;10:9453. doi: 10.1038/s41598-020-66410-1 (PMC7289851; doi:10.1038/s41598-020-66410-1)
Supplement: Supplementary file 2 — Supplementary information2. [file 41598_2020_66410_MOESM2_ESM.pdf]

Enhanced rate capabilities in a glass-ceramic-derived sodium all-solid-state battery

Hideo Yamauchi[1], Junichi Ikejiri[1], Kei Tsunoda[1], Ayumu Tanaka[1], Fumio Sato[1], Tsuyoshi Honma[2*], Takayuki Komatsu[2]

[1]Nippon Electric Glass Co., Ltd., Shiga 520-8639, Japan
[2]Department of Materials Science and Technology, Nagaoka University of Technology, Kamitomioka-cho 1603-1, Nagaoka, Niigata 940-2188, Japan

*Correspondence: Tsuyoshi Honma
e-mail
honma@mst.nagaokaut.ac.jp

Table S1. Properties of all-solid-state batteries (ASSBs) examined in this study: specific surface areas of $Na_2FeP_2O_7$ (NFP) and β"-alumina, average particle size, calcination temperature and time, composition of the cathode layer, weight of cathode materials, thickness of the cathode layer, and cell capacity.

Cell	Powder		Calcination condition	Cathode						Cell capacity (as NFP:80mAh/g) (mAh/cm$^{2)}$
	BET-SSA (m^2/g) (Average particle size (μm))			Composition (wt%)			Cathode materials (mg)	Thickness (μm)	NFP loading (mg)	
	NFP	β"-Alumina		NFP	β"-Alumina	Acetylene black				
A	10 (0.8*)	5 (2*)	550°C-1hour	72	25	3	5.41	30	3.9	0.31
B	27 (0.2**)	45 (0.1**)	525°C-0.5hour	83	13.5	3.5	4.2	25	3.49	0.28
C	27 (0.2**)	45 (0.1**)	525°C-0.5hour	83.4	12.4	4.2	3.79	22	3.16	0.25
D	27 (0.2**)	45 (0.1**)	525°C-0.5hour	83.4	12.4	4.2	5.41	40	4.51	0.36
E	27 (0.2**)	45 (0.1**)	525°C-0.5hour	83.4	12.4	4.2	4.41	30	3.68	0.36***
Ref. 1M $NaPF_6$ in EC/DEC	N/A(1.7*)	–	–	90	5(PVDF)	5	6.88	40	6.19	0.6

*Laser diffraction particle size diffraction analysis, ** Analysis of SEM images, *** Cell capacity as 97mAh/g of NFP

Table S2. Analysis of the Nyquist plot of Cell A.

Battery components	Materials			Composites		Symmetric cells		All-solid-state Na ion battery
Cathode (t35μm)	$Na_2FeP_2O_7$ glass ceramic (calc. as thickness 35μm)	R_{total}	**35000Ω**	R_{total} **0.1Ω**	R_{total} **31000Ω**	$R_{cathode}$ 27000Ω		**38773Ω**
		R_{bulk}	N/A		R_{NFP} 9000Ω			
		$R_{grain\ boundary}$	N/A		$R_{NFP\text{-}SE\ powder}$ 22000Ω			
	Acetylene black (calc. as thickness 35μm)	R	1.1Ω					
	β"-Alumina powder (calc. as thickness 2μm)	R_{total}	0.14Ω					
		R_{bulk}	0.05Ω					
		$R_{grain\ boundary}$	0.09Ω					
Interface						$R_{interface}$ **2350Ω**		**2571Ω**
Solid electrolyte (t1mm)	β"-Alumina	R_{total}	**72Ω**			R_{total} 73Ω	R_{total} 72Ω	**381Ω**
		R_{bulk}	27Ω			R_{bulk} 25Ω	R_{bulk} 27Ω	
		$R_{grain\ boundary}$	45Ω			R_{gb} 48Ω	R_{gb} 45Ω	
Interface							$R_{interface}$ **125Ω**	
Anode (t35μm)	Na metal	R	**1.0Ω**				R_{anode} 20Ω	**65Ω**

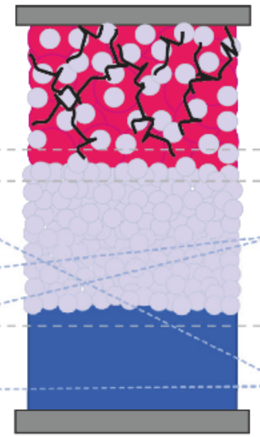

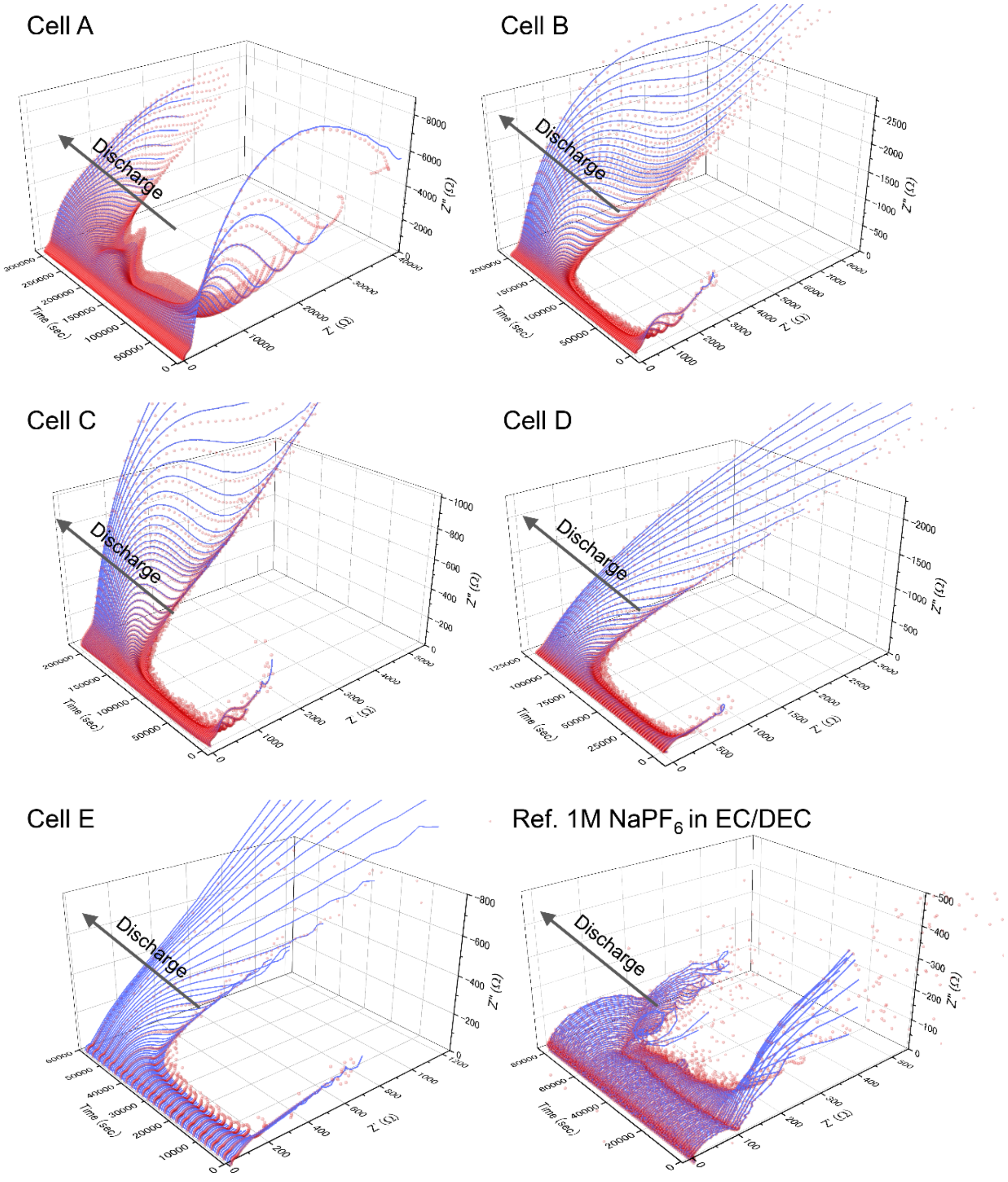

Figure S1. Three-dimensional (3D) impedance spectra of Cells A–E and 1 M $NaPF_6$ in EC/DEC acquired during the discharge process.

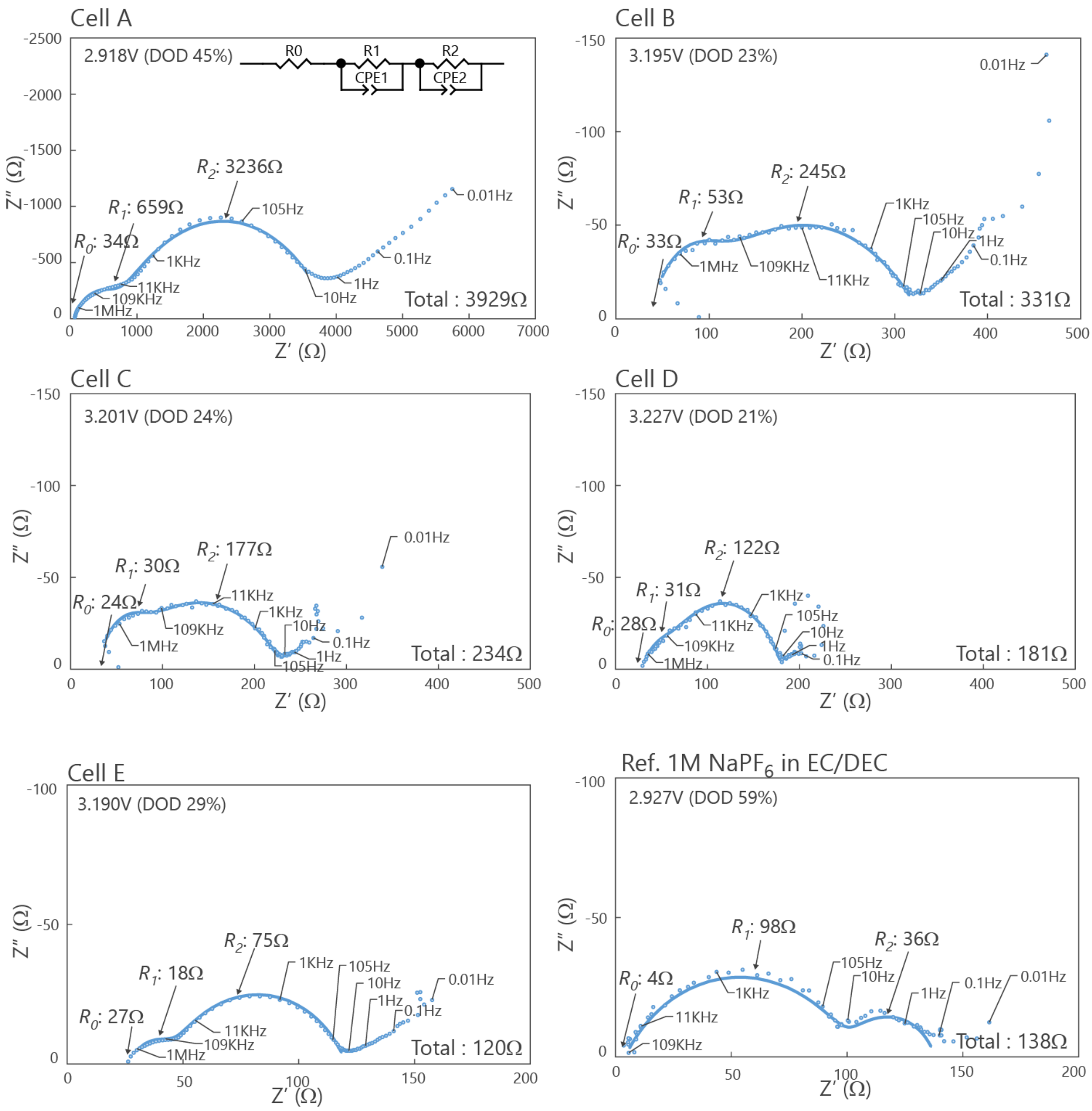

Figure S2. Nyquist plots of Cells A– E and 1 M $NaPF_6$ in EC/DEC obtained from 3D impedance spectra (Fig. S1).

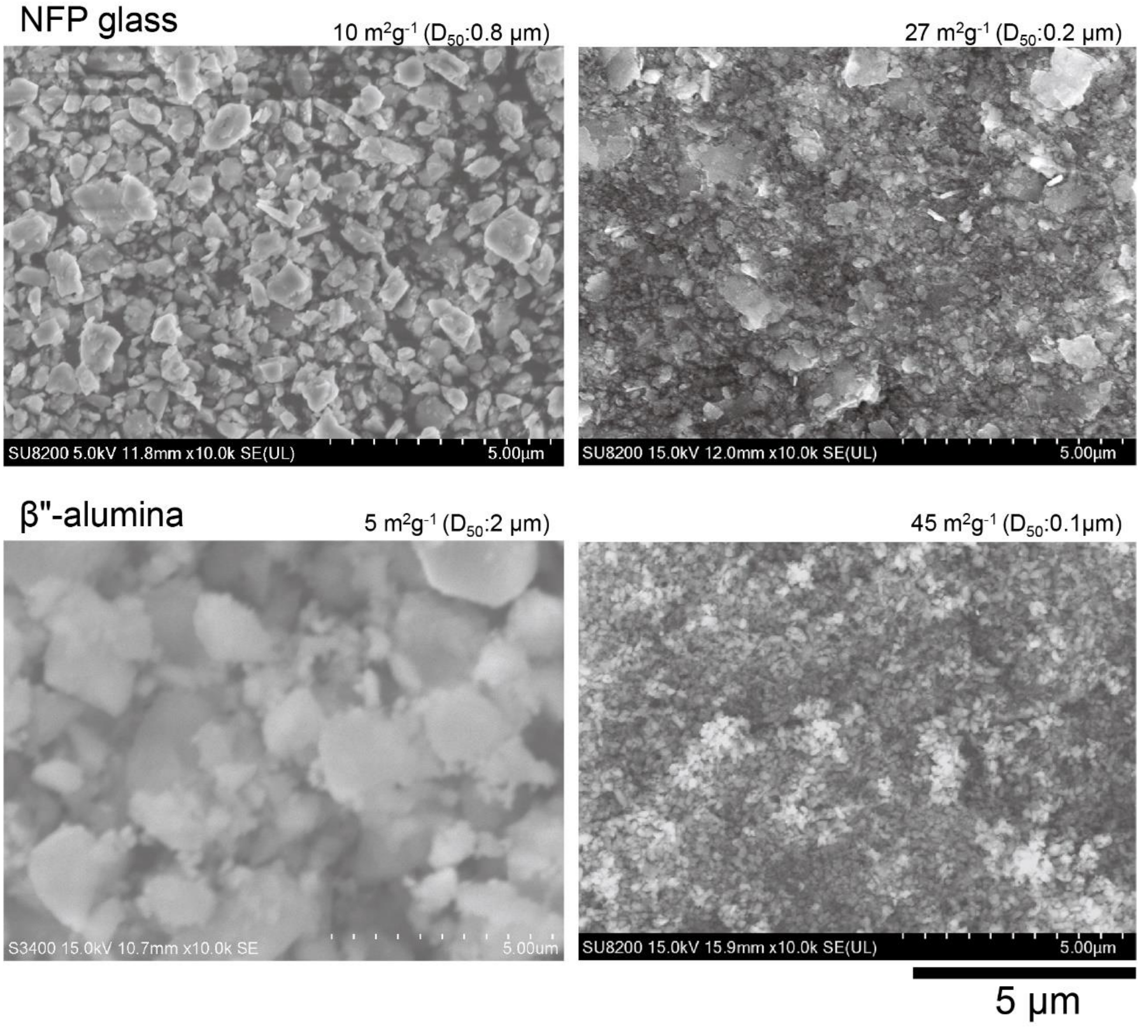

Figure S3. SEM images of NFP glass powder and β"-alumina solid solution.

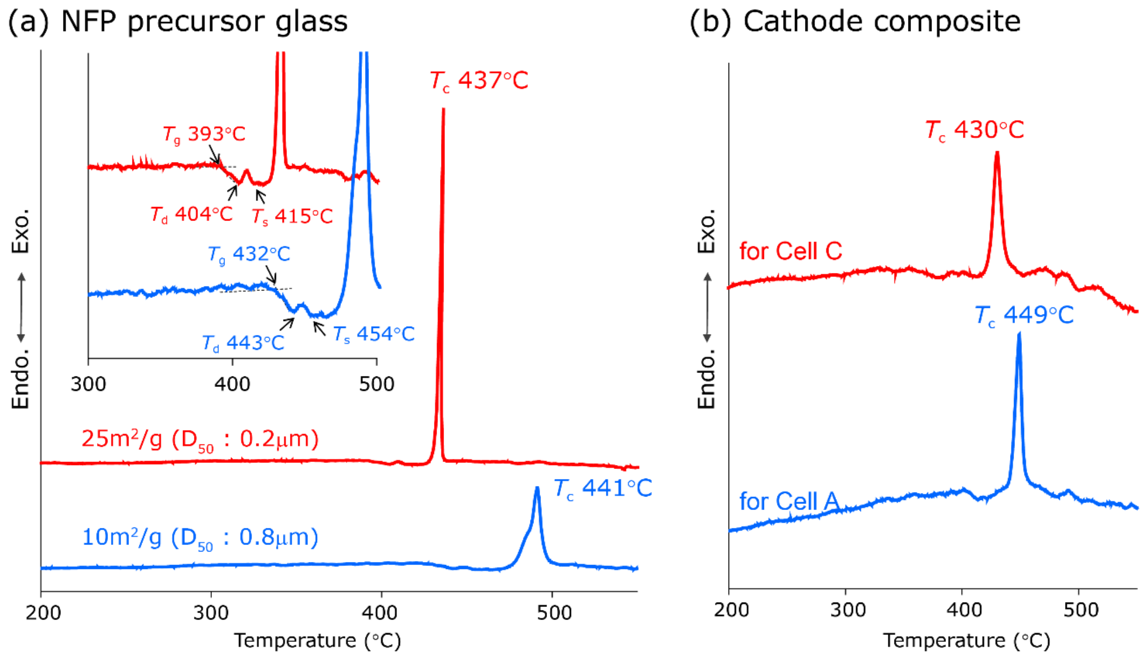

Figure S4. Differential thermal analysis curves of NFP glasses with different specific surface areas. T_d represents the yield point and T_s represents the softening point.

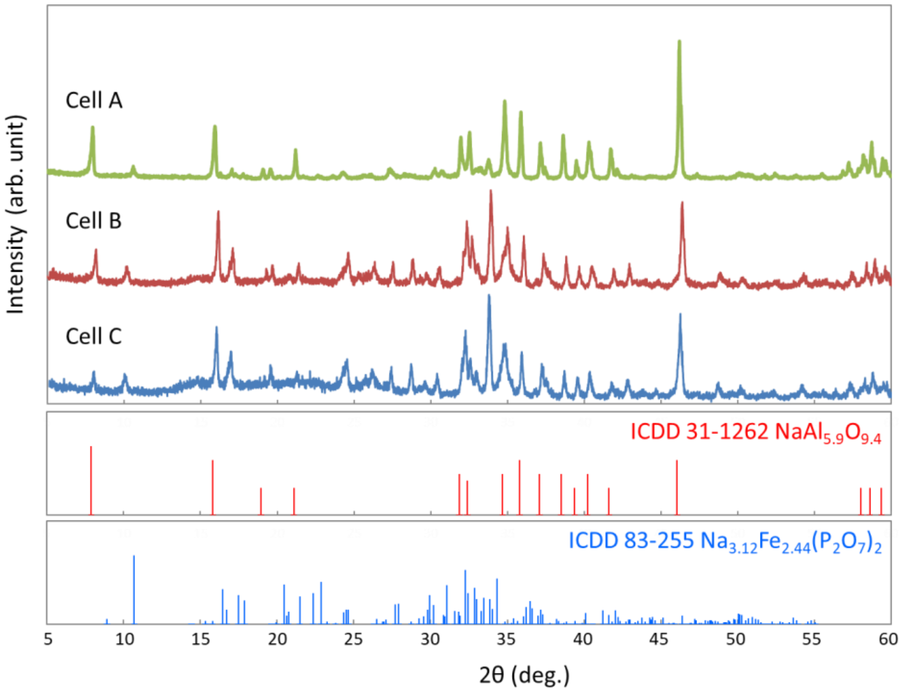

Figure S5. X-ray diffraction patterns of the NFP glass-ceramic cathode on β"-alumina solid electrolyte substrate calcined at 550 °C for 60 min (Cell A) and 525 °C for 30 min (Cells B and C) in H_2/N_2 (4/96 v/v%). Cathode composition, NFP glass-ceramic : β"-alumina : AB = 72 : 25 : 3 wt% (Cell A), 83 : 13.5 : 3.5 wt% (Cell B), 83.4 : 12.4 : 4.2 wt% (Cell C).

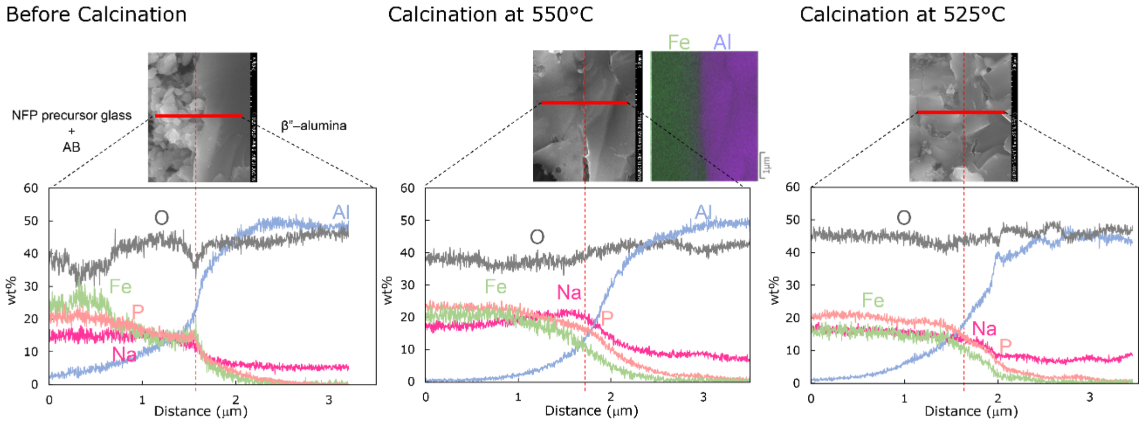

Figure S6. Scanning electron microscopy images of the NFP–β"-alumina interface and linear analysis profiles of components. The observations were made at an acceleration voltage of 15 kV and 25000× magnification.

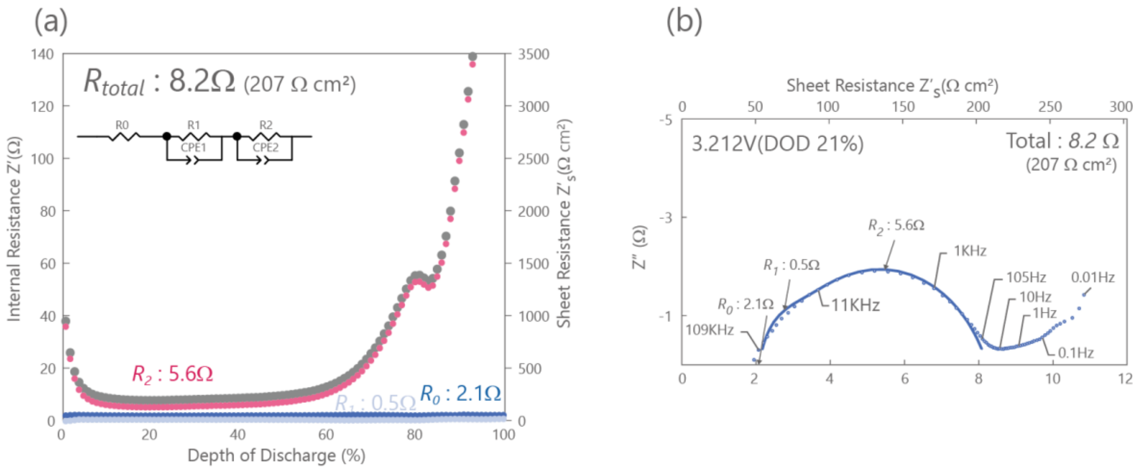

Figure S7. (a) Dependence of the internal resistance Z' (Ω) and sheet resistance Z_s' (Ω cm^2) of the pouch cell based on Cell C on the depth of discharge, (b) Nyquist plot of the pouch cell obtained from 3D impedance spectra.

Supplementary video

Electric fan powered by the $Na_2FeP_2O_7$ glass ceramic cathode|β"-alumina|Na pouch cell sandwiched between two ice blocks.
